# Quiz Compends on Physiology

**Published:** 1892-04

**Authors:** 


QUIZ COMPENDS.—Physiology, by Dr. Brubake. Published by P. Blakiston, Son & Co., Philadelphia.

This compend on physiology is now issued as the the sixth edition of the work, which is an evidence of its popularity among students and practitioners. It is often impossible for practitioners of medicine to read thoroughly an extended treatise on various subjects, in order to be posted on the very latest advances in medicine, and for this reason, late editions of compends are of great value as such. This last edition is very thorough and complete. C. D. R.

